# Stellate Ganglion Block Combined With Subanesthetic Ketamine Versus Dexmedetomidine for the Management of Paroxysmal Sympathetic Hyperactivity (PSH) After Traumatic Brain Injury: A Randomized Trial

**DOI:** 10.1155/ccrp/2779975

**Published:** 2026-11-04

**Authors:** Eslam Sobhy Al Maghawry Mohamed, Alshaimaa Abdel Fattah Kamel, Abdalla Mohamed Goda Mohamed, Abd El-Rahman Wael Abd El-Rahman Ali Elmesallamy, Mohamed Gaber Mohamed, Michael Adel Shaker, Omar A. Khashaba, Marwa Mohamed Medhat

**Affiliations:** ^1^ Anesthesia Intensive Care and Pain Management Department Faculty of Medicine Zagazig University Alsharkia Egypt zu.edu.eg; ^2^ Faculty of Human Medicine Zagazig University Zagazig Alsharkia Egypt zu.edu.eg; ^3^ Dubai Hospital Dubai UAE dha.gov.ae; ^4^ Radiology Department Faculty of Medicine, Zagazig University Alsharkia Egypt

**Keywords:** dexmedetomidine, ketamine, paroxysmal sympathetic hyperactivity, stellate ganglion block, traumatic brain injury

## Abstract

**Background:**

With appropriate treatment of paroxysmal sympathetic hyperactivity (PSH) following a traumatic brain injury, there is hope for individuals to overcome storming, regain consciousness, and progress toward successful recovery from the brain injury. The primary goal was to monitor changes in mean arterial blood pressure (MABP) in TBI patients with PSH treated with stellate ganglion block combined with subanesthetic dose ketamine versus dexmedetomidine infusion. The secondary outcomes included monitoring changes in heart rate, respiratory rate, temperature decline, duration of the resolution of paroxysmal hypermyotonia, Glasgow Coma Scale (GCS) grading, intensive care unit (ICU) stay, and the number of patients who did not show improvement.

**Methods:**

This randomized, double‐blind controlled study was conducted on 102 patients with traumatic brain injury experiencing PSH. They were randomly allocated into two groups: the SK group (*n* = 51), where patients received a unilateral stellate ganglion block with 5 mL of bupivacaine 0.5% combined with 0.5 mg/kg/h intravenous subanesthetic ketamine infusion, and the D group (*n* = 51), where patients received a loading dose of 1 μg/kg dexmedetomidine for 10 min and then starting an intravenous infusion at a rate of 1 μg/kg/h.

**Results:**

The D group exhibited a significant earlier decrease in MABP compared to the SK group at 30 min, 1, 2, 4, 8, 16, 24, and 48 h (*p* value = 0.0001). Both groups experienced similar reductions in RR at the same time points (*p* values ≥ 0.5). The temperature decreased after approximately 10 h from initiating treatment with no significant difference between the two groups. Four patients in the D group and three patients in the SK group did not show significant improvement. In terms of GCS, patients in both groups demonstrated significant improvement in GCS grading (18 [35.3%] Grade I, 29 [56.9%] Grade II, and only 1 [2.0%] and 3 [5.9%] Grades III and IV, respectively, in the D group, and 20 [39.2%] Grade I, 28 [54.9%] Grade II, and only 2 [3.9%] Grade III and 1 [2.0%] Grade IV in the SK group) (*p* = 0.0001). Both groups had similar durations of ICU stay (*p* = 0.222).

**Conclusions:**

A unilateral stellate ganglion block combined with subanesthetic ketamine improves overall neurological recovery and ICU length of stay in post‐TBI PSH compared to continuous dexmedetomidine. However, dexmedetomidine provides a faster reduction in MABP and offers high practical value due to its noninvasive, intravenous delivery.

**Trial Registration:** ClinicalTrials.gov identifier: NCT06354673.

## 1. Introduction

Paroxysmal sympathetic hyperactivity (PSH), colloquially known in older literature as a “neurostorm,” is an intense autonomic stress reaction that occurs after a traumatic brain injury (TBI) and can persist for weeks or months. It involves periodic episodes of unprovoked tachycardia, severe hypertension, diaphoresis, hyperthermia, and rigid motor posturing, often triggered by benign external stimuli. As emphasized by Krupanandan, moving away from ambiguous descriptions like “neurostorming” toward standardized, criteria‐backed PSH terminology is vital for establishing accurate diagnostic baselines and guiding targeted medical therapies across neurocritical care centers [1].

PSH has been reported in up to 33% of adult patients suffering from severe TBI and can be a life‐threatening condition [2]. Proper control of blood pressure is vital in the treatment of PSH as uncontrolled hypertension can lead to poorer outcomes for the patient. Ultimately, timely identification and suitable treatment approaches are crucial for lessening the effects of PSH on recovery and enhancing patient outcomes [3].

The stellate ganglion block (SGB) is a treatment primarily used for pain‐related diseases. A human study has shown that unilateral SGB can increase cerebral blood flow in one hemisphere of the brain. This led us to boldly assume that SGB may play a role in improving cerebral circulation and reducing secondary brain injury in patients with TBI [4].

The stellate ganglion is formed by the fusion of the sympathetic ganglia at C7 and T1, and it is the location where a cervical sympathetic block (CSB) is applied. Even though in 17% of the population there is no fusion at all from a strictly anatomical perspective, a cervical sympathetic trunk block is still commonly referred to as a SGB in many studies. These studies have shown that SGB can prevent and alleviate cardiovascular symptoms by inhibiting the cervical sympathetic nerves from dilating brain blood vessels and increasing cerebral blood flow, thereby improving microcirculation in the brain and having a certain protective effect on the brain [5, 6].

Subanesthetic ketamine infusion (KI) has been shown to increase neuroplasticity and memory improvement and CSB has been shown to improve cognitive function and reduce sympathetic overactivity [7]. Subanesthetic KI is known to alter hippocampal cell proliferation following TBI, which is a possible benefit from KI following TBI in mice [8]. Dr. Browne reported that ketamine was effective for treating complications that emerge after blast injury [9].

Dexmedetomidine, a highly selective alpha‐2 adrenergic agonist, has shown a clear ability to reduce sympathetic activation. Emerging evidence suggests dexmedetomidine may provide neuroprotective effects, potentially reducing secondary brain injury. However, more research is needed to confirm long‐term outcomes [10]. Recently, a milestone randomized controlled trial by Wang et al. demonstrated that ultrasound‐guided right‐sided stellate ganglion blocks (RSGBs) could safely and significantly reduce the severity scores (CFS/PSH‐AM) and total duration of acute PSH attacks in acquired brain injury by directly reducing circulating plasma epinephrine and norepinephrine levels [11]. While Wang et al. established the safety of SGB as an adjuvant added to conventional care, there remains a critical gap in literature comparing SGB‐based regimens head‐to‐head against established continuous first‐line antisympathetic drug infusions. The purpose of this study was to evaluate the comparative effect of an SGB‐centric approach combined with a subanesthetic KI versus standard dexmedetomidine infusion.

## 2. Methods

### 2.1. Study Design and Population

This prospective randomized comparative double‐blind clinical study was conducted in the surgical intensive care unit at Academic University Hospitals between April 2024 and September 2025. The institutional review board of the Faculty of Medicine at Zagazig University approved this trial with the reference number ZU‐IRB# 278‐1‐4‐2024.

The study focused on critically ill patients with traumatic brain injuries who suffered from PSH, diagnosed based on the criteria proposed by Baguley et al. [12] (Supporting Table 1). The participants were aged 18 years or older, of both sexes, with a body mass index of 35 kg/m^2^ or less. All patients enrolled in this trial presented with closed severe TBI (postresuscitation admission GCS score 4–8) resulting primarily from high‐velocity motor vehicle accidents or accidental falls from heights. Concurrent stable nonhemorrhagic extracranial trauma. The average time elapsed from the primary TBI to the diagnosis of PSH and subsequent trial enrollment was approximately 7 days. Informed written consent was obtained from the first‐degree relative or legal guardian before enrollment in the study.

The primary outcome of the study was to document changes in mean arterial blood pressure (MABP) at 30 min, 1, 2, 4, 8, 16, 24, and 48 h after starting treatment in TBI patients with PSH who were treated with SGB combined with either subanesthetic dose KI versus dexmedetomidine infusion. Secondary outcomes included monitoring changes in heart rate (HR) and respiratory rate at the same time points, assessing temperature during the episode, measuring the duration of temperature decrease, duration of resolution of paroxysmal hypermyotonia, evaluating changes in Glasgow Coma Scale (GCS) score before and after treatment, length of ICU stay, and documenting the number of patients who did not show improvement.

The patients and the anesthesiologist assessing the primary and secondary outcomes were blinded to the interventional groups in the study.

This study excluded individuals with primary brain stem damage or hemorrhage, as well as those with a GCS score of 3 (Supporting Table 2) [13]. Additionally, patients were not included if they had experienced intracerebral hemorrhage or infarction within the previous 3 months, had terminal cancer, uncontrolled epilepsy, severe blood clotting disorders, hemorrhagic shock, multiorgan failure, serious systemic organ diseases, or known allergies to the medications used in the study.

Patients underwent a comprehensive evaluation which included a detailed medical history, physical examination, and various laboratory tests such as complete blood count, coagulation profile, and assessments of liver and kidney function. During the physical assessment, particular emphasis was placed on recording basal MABP in mmHg, HR in beats/min, respiratory rate (RR), temperature in degrees Celsius, and grading of GCS before initiating treatment.

The grading GCS criteria for patients [14] enrolled in the study were in relation to the patient’s GCS [13].

 Grade 0 = GCS score of 15
 Grade 1 = GCS score of 15
 Grade 2 = GCS score of 13–14
 Grade 3 = GCS score of 9–12
 Grade 4 = GCS score of 3–8

### 2.2. Allocation Concealment and Blinding

All patients meeting the inclusion criteria were randomly allocated into two equal groups. Randomization sequences were generated via a computer and concealed using sequentially numbered, opaque, sealed envelopes (SNOSE). An independent ICU nurse, who was entirely isolated from data collection and patient care, opened the envelopes and prepared the study interventions. Dexmedetomidine infusions and the matching subanesthetic ketamine/saline combinations were prepared in identical, unlabeled 50 mL syringes to maintain strict blinding of both the active treating clinicians and the outcome assessors.

*Ketamine group (SK group):* Patients received unilateral SGB with 5 mL of bupivacaine 0.5%. Combined with an intravenous infusion of ketamine at a rate of 0.5 mg/kg/h.

*Dexmedetomidine group (D group):* Patients received a loading dose of 1 μg/kg dexmedetomidine for 10 min then starting an intravenous infusion at a rate of 1 μg/kg/h.

All patients received standardized neuroprotective ICU care including head‐of‐bed elevation (30°), strict normothermia control, a single‐occupancy darkened ICU room, strict noise limits (< 40 dB), clustered minimal‐handling nursing care, rigorous routine bowel and bladder management, and standard baseline sedation with 0.5–2 mg/kg/h propofol when mechanically ventilated and for preventing severe agitation. One to two milligrams IV bolus midazolam was restricted to refractory breakthrough seizures or severe ventilator dyssynchrony. Pharmacological adjuncts utilized as institutional standard of care for maintenance therapy included scheduled intravenous paracetamol 1 g every 6 h and scheduled enteral propranolol (20–40 mg every 8 h), which were kept constant throughout the 48‐h study window. Any adverse effects such as bradycardia, hypotension requiring vasopressors, respiratory depression, and block‐related complications were detected and treated.

### 2.3. Performance of SGB

The patient was placed in the supine position with the head slightly rotated in the opposite direction. The skin of the neck was cleaned with an alcohol preparation, and 2 g of sterile ultrasound gel were applied. An ultrasound machine (GE Ultrasound Korea, Ltd.) with a high‐frequency linear transducer (8–12 MHz) was used.

To view the C6 nerve root from the interscalene plexus to the foramen, which is flanked by the prominent anterior tubercle and short posterior tubercle, the transducer was positioned cephalad and lateral. Local anesthetic (5 mL of 0.5% bupivacaine) was then injected beneath the prevertebral fascia covering the longus colli muscle using an in‐plane method from lateral to medial (Figure 1). To prevent dissemination along the carotid sheath, the needle tip should be positioned anterolateral to the longus colli muscle and deep to the prevertebral fascia. It should also be superficial to the muscle fascia to avoid intramuscular injection [15].

**FIGURE 1:**
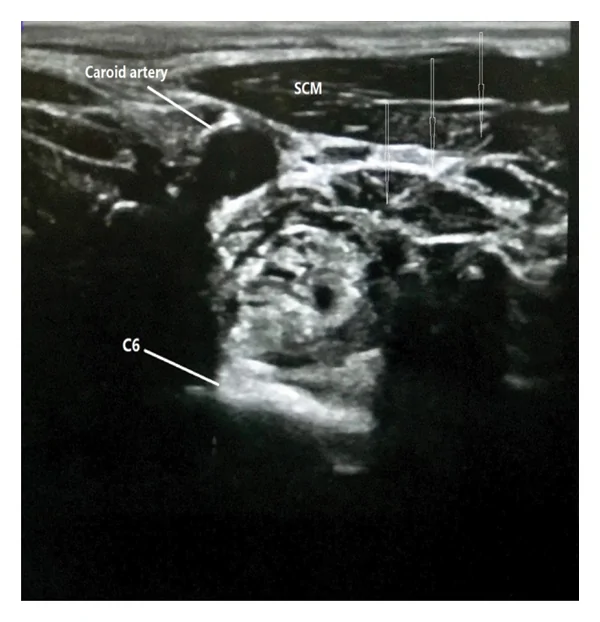
Needle trajectory for stellate ganglion block. SCM = sternoclenomastoid. Arrows point to the needle.

After the relief of PSH, the duration of resolution of paroxysmal hypermyotonia (defined as the duration from the start of treatment until resolution) and grading of GCS after the relief of PSH were recorded. Additionally, the duration of ICU stay (the duration from admission until discharge from ICU) and the number of patients who did not show any improvement were also recorded.

### 2.4. Statistical Analysis

Based on a pilot study, the sample size was determined using G Power version 3.1.9.7. A moderate effect size was expected between the SGB combined to ketamine group (SK group) and dexmedetomidine group (D group) (*d* = 0.5). With a confidence interval of 95% and a power of 80%, the sample size was calculated to be 102, or 51 for each group.

Data collection and analysis were conducted using the Statistical Package for the Social Sciences (SPSS) version 23.0 software, following a per‐protocol analysis. Qualitative variables were presented as frequencies and percentages, while quantitative data were presented as mean ± SD, and range. Parametric tests were utilized to compare groups when the data were normally distributed. The *t* test was used for comparing two groups with quantitative normally distributed variables, while the chi‐square test (*χ* ^2^) was used to compare different qualitative variables. The marginal homogeneity test was conducted to study paired ordinal variables. All tests were two‐sided, with a *p* value  < 0.05 considered statistically significant, and a *p* value  ≥ 0.05 considered statistically insignificant. Repeated‐measures ANOVA was used to evaluate MAP changes over time and differences between groups. As sphericity was violated, the Greenhouse–Geisser correction was applied. Time, group, and time‐by‐group interaction effects were assessed, with *p* < 0.05 considered statistically significant. Bonferroni‐adjusted pairwise comparisons.

## 3. Results

During our study period, 110 critically ill patients with TBI who suffered from PSH were eligible for enrollment. Eight patients were excluded as they did not meet inclusion criteria (three patients suffered uncontrolled epilepsy, two patients experienced intracerebral hemorrhage, and three patients with GCS 3). Therefore, 102 TBI patients suffering from PSH were included and randomly allocated into two equal groups (Figure 2).

**FIGURE 2:**
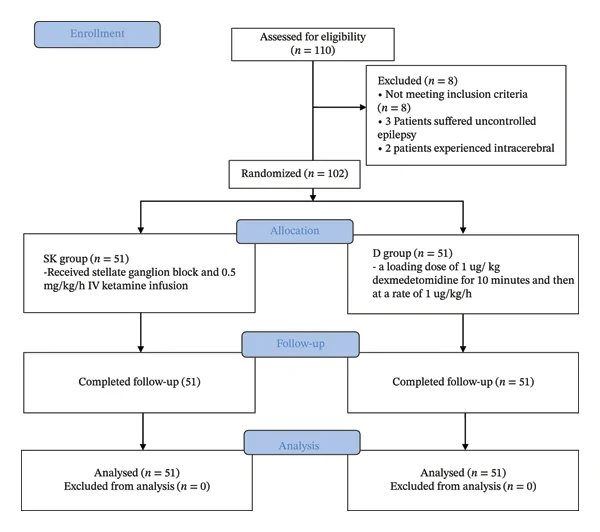
Consort flow diagram *n* = number of patients, IV = intravenous. Glasgow Coma Scale (GCS).

The clinical features of the patients, including age, sex, BMI, and ASA PS I and II, did not show a statistically significant difference between the two groups studied (*p* value ≥ 0.5) (Table 1).

**TABLE 1:** Patients’ characteristics.

Characteristics	Group SK (*n* = 51)	Group D (*n* = 51)	*p*value
Age (years)	33.06 ± 10.15	32.39 ± 10.48	0.745
BMI (kg/m^2^)	28.71 ± 2.09	27.96 ± 2.33	0.095
Sex *N* (%)			
Females	21 (41.2%)	19 (37.3%)	0.685
Males	30 (58.8%)	32 (62.7%)	
ASA PS, *N* (%)			
I	28 (54.9%)	31 (60.8%)	0.547
II	23 (45.1%)	20 (39.2%)	

*Note:* Group SK = Stellate ganglion with ketamine group, Group D = dexmedetomidine group, *N* = total number of subjects in each group. Quantitative data are expressed as mean ± standard deviation (SD). Qualitative data as number and percent.

Independent samples Student′s *t* test. *χ* ^2^, chi‐square test.

^∗^ *p* value < 0.05: significant.

There was no significant difference in basal MABP and HR between the groups before treatment initiation. However, the D group showed a statistically significant earlier decrease in MABP compared to the SK group at 30 min, 1, 2, 4, 8, 16, 24, and 48 h (*p* value = 0.0001) (Table 2).

**TABLE 2:** Mean arterial blood pressure (MABP) (mmHg) between the studied groups at the measured time points.

MABP	Group SK (*n* = 51)	Group D (*n* = 51)	*p* value
Basal (before initiating treatment)	115.49 ± 3.48	114.76 ± 3.52	0.298
At 30 min after initiating treatment	110.76 ± 2.92	99.08 ± 4.07	0.0001
At 1 h after initiating treatment	108.18 ± 3.15	95.12 ± 2.95	0.0001
At 2 h after initiating treatment	103.19 ± 2.91	67.55 ± 4.55	0.0001
At 4 h after initiating treatment	78.18 ± 2.63	63.98 ± 2.40	0.0001
A 8 h after initiating treatment	75.24 ± 2.29	62.27 ± 1.63	0.0001
At 16 h after initiating treatment	72.29 ± 1.95	61.09 ± 1.25	0.0001
At 24 h after initiating treatment	69.82 ± 1.23	61.18 ± 1.24	0.0001
At 48 h after initiating treatment	70.57 ± 0.92	60.76 ± 0.81	0.0001

*Note:* Group SK = Stellate ganglion with ketamine group, Group D = dexmedetomidine group, *n* = total number of subjects in each group. Quantitative data are expressed as mean ± standard deviation (SD). Independent samples Student′s *t* test.

^∗^ *p* value < 0.05: significant.

There was a statistically significant change in mean arterial pressure (MAP) over time (*p* < 0.001, partial *η* ^2^ = 0.987). A significant difference was also observed between the two groups (*F* = 1829.086, *p* < 0.001, partial *η* ^2^ = 0.948), with a significant group‐by‐time interaction (*F* = 420.266, *p* < 0.001, partial *η* ^2^ = 0.808), indicating that MAP changes over time differed significantly between groups. MAP was comparable at baseline between Group SK and Group D (*p* > 0.05), After that, MAP remained significantly higher in Group SK at all follow‐up measurements, with upmost difference value at 2 h. Bonferroni‐adjusted pairwise comparisons showed statistically significant differences between all assessment time points (*p* < 0.001) (Table 3).

**TABLE 3:** Change in mean arterial pressure (MAP) over time.

Effect	*F*	*p* value	Partial *η* ^2^
MAP (time effect)	7414.261	< 0.001	0.987
Group	1829.086	< 0.001	0.948
MAP × Group interaction	420.266	< 0.001	0.808

There was no significant difference in basal HR between the groups before treatment initiation. However, the D group showed a statistically significant earlier decrease in HR compared to the SK group at 30 min, 1, 2, 4, 8, 16, 24, and 48 h (*p* value = 0.0001) (Table 4).

**TABLE 4:** Heart rate (HR) (beat/min) between the studied groups at the measured time points.

HR	Group SK (*n* = 51)	Group D (*n* = 51)	*p* value
Basal (Before initiating treatment)	135.96 ± 2.62	135.45 ± 2.67	0.333
At 30 min after initiating treatment	97.37 ± 2.12	72.92 ± 1.89	0.0001
At 1 h after initiating treatment	93.29 ± 2.77	71.08 ± 1.69	0.0001
At 2 h after initiating treatment	74.27 ± 2.88	69.94 ± 1.53	0.0001
At 4 h after initiating treatment	72.37 ± 2.57	65.76 ± 1.41	0.0001
At 8 h after initiating treatment	70 ± 2.61	62.37 ± 2.48	0.0001
At 16 h after initiating treatment	68.96 ± 2.54	62.25 ± 2.24	0.0001
At 24 h after initiating treatment	67.27 ± 2.35	61.24 ± 1.56	0.0001
At 48 h after initiating treatment	66.12 ± 2.19	61.12 ± 1.61	0.0001

*Note:* Group SK = Stellate Ganglion with Ketamine group, Group D = Dexmedetomidine group, *n* = Total number of subjects in each group. Quantitative data are expressed as mean ± standard deviation (SD). Independent samples Student′s *t* test.

^∗^ *p* value < 0.05: significant.

There was a statistically significant change in HR over time (*p* < 0.001, partial *η* ^2^ = 0.993). A significant difference was also observed between the two groups (*p* < 0.001, partial *η* ^2^ = 0.919), with a significant group‐by‐time interaction (*p* < 0.001, partial *η* ^2^ = 0.830), indicating that HR changes over time differed significantly between groups. HR was comparable at baseline between Group SK and Group D. Thereafter, HR differed significantly between groups at most follow‐up measurements, with the greatest between‐group differences observed from 30 min onward. Bonferroni‐adjusted pairwise comparisons showed statistically significant differences between almost all assessment time points (*p* < 0.001), except between 8 and 16 h (*p* = 0.310) (Table 5).

**TABLE 5:** Change in heart rate (HR) over time.

Effect	*F*	*p* value	Partial *η* ^2^
Heart rate (time)	15036.234	< 0.001	0.993
Group	1141.772	< 0.001	0.919
Heart rate × Group	486.879	< 0.001	0.830

Regarding respiratory rate (RR), both groups showed comparable lowering of RR at 30 min, 1, 2, 8, 16, 24 and 48 h after initiating treatment (*p* values ≥ 0.5) (Table 6).

**TABLE 6:** Respiratory rate (RR) between the studied groups at the measured time points.

RR	Group SK (*n* = 51)	Group D (*n* = 51)	*t*	*p* value
Basal (Before initiating treatment)	42.43 ± 1.95	42.61 ± 1.93	0.459	0.647
At 30 min after initiating treatment	39.82 ± 1.85	39.12 ± 1.86	1.920	0.058
At 1 h after initiating treatment	37.37 ± 2.08	38.04 ± 1.64	1.800	0.075
At 2 h after initiating treatment	34.06 ± 2.61	34.22 ± 1.84	0.351	0.726
At 4 h after initiating treatment	24.18 ± 2.46	23.51 ± 1.58	1.631	0.106
At 8 h after initiating treatment	22.02 ± 2.41	21.71 ± 1.42	0.801	0.425
At 16 h after initiating treatment	20.53 ± 2.11	20.18 ± 0.99	1.080	0.283
At 24 h after initiating treatment	17.73 ± 1.87	17.45 ± 1.21	0.882	0.380
At 48 h after initiating treatment	15.28 ± 1.64	15.55 ± 1.24	0.955	0.342

*Note:* Group SK = Stellate ganglion with ketamine group, Group D = dexmedetomidine group, *n* = total number of subjects in each group. Quantitative data are expressed as mean ± standard deviation (SD). Independent samples Student′s *t* test.

^∗^ *p* value < 0.05: significant.

Respiratory rate demonstrated a highly significant reduction over time (*p* < 0.001, partial *η* ^2^ = 0.984). No significant difference between Group SK and Group D (*p* = 0.672, partial *η* ^2^ = 0.002). However, the significant time‐by‐group interaction (*F* = 3.026, *p* = 0.020, partial *η* ^2^ = 0.029) indicated that the pattern of respiratory rate change over time differed significantly between the two groups. Pairwise comparisons showed significant differences between all respiratory rate time points after Bonferroni adjustment (all *p*s < 0.001). Confirming a progressive change in respiratory rate over the follow‐up (Table 7).

**TABLE 7:** Change in respiratory rate (RR) over time.

Effect	*F*	*p* value	Partial *η* ^2^
Respiratory rate (time)	6095.539	< 0.001	0.984
Group	0.180	0.672	0.002
Respiratory rate × Group	3.026	0.020	0.029

There was no notable difference between the two groups in terms of basal (before initiating treatment) temperature. However, the temperature declined after approximately 10 h from initiating treatment, with no statistically significant difference in its subsequent decline between the two groups (Table 8).

**TABLE 8:** Degree of temperature before initiating treatment, duration of decline of temperature, duration paroxysmal hypermyotonia (days) and number of patients not improved.

Variables	Group SK (*n* = 51)	Group D (*n* = 51)	*p* value
Basal (degree of temperature before initiating treatment) (C°)	40.60 ± 0.434	40.43 ± 0.48	0.066
Duration of decline of temperature after initiating treatment (hours)	10.80 ± 1.00	10.65 ± 1.16	0.467
Duration of resolution of paroxysmal hypermyotonia (days)	9 ± 1.44	8.71 ± 0.9	0.102
Duration of ICU stay (days)	12.56 ± 1.96	12 ± 1.45	0.220
Number of patients not improved, *N* (%)	3 (5.9%)	4 (7.8%)	0.99

*Note:* Group SK = Stellate ganglion with ketamine group, Group D = dexmedetomidine group, *n* = total number of subjects in each group. Quantitative data are expressed as mean ± standard deviation (SD). Independent samples Student′s *t* test. Fisher’s exact test.

Abbreviation: ICU, intensive care unit.

^∗^ *p* value < 0.05: significant.

The duration of resolution of paroxysmal hypermyotonia and ICU stay were comparable between the two groups. There were four patients in the D group compared to three patients in the SK group who did not show significant improvement, and both groups were comparable (Table 8).

Regarding GCS, patients in both groups had Grades III and IV GCS before initiating treatment (19 [37.3%] Grade III and 32 [62.7%] Grade IV in the D group compared to 22 [43.1%] Grade III and 29 [56.9%] Grade IV in the SK group) with no statistically significant difference between the two groups before improvement (*p* = 0.545). However, after improvement, patients showed a statistically and clinically significant improvement in GCS grading within the same group (18 [35.3%] Grade I, 29 [56.9%] Grade II, and only 1 [2.0%] and 3 [5.9%] Grades III and IV, respectively, in the D group and 20 [39.2%] Grade I, 28 [54.9%] Grade II, and only 2 [3.9%] Grade III and 1 [2.0%] Grade IV in the SK group) (*p* = 0.0001) (Table 9).

**TABLE 9:** GCS grade between the studied groups.

Variables	Group SK (*n* = 51)	Group D (*n* = 51)	*p* value
Basal GCS grading (before initiating treatment)			
Grade III	22 (43.1)	19 (37.3)	0.545
Grade IV	29 (56.9)	32 (62.7)	
GCS grading (after improvement)			
Grade I	20 (39.2)	18 (35.3)	0.692
Grade II	28 (54.9)	29 (56.9)	
Grade III	2 (3.9)	1 (2.0)	
Grade IV	1 (2.0)	3 (5.9)	
^H^P	0.0001^∗^	0.0001^∗^	

*Note:* *χ* ^2^, chi‐square test, H: marginal homogeneity test. *p* value ≥ 0.05: not significant.

^∗^ *p* value < 0.05: significant.

## 4. Discussion

To the best of our knowledge, this is the first randomized clinical trial that evaluate the comparative effects of SGB combined with subanesthetic dose ketamine versus dexmedetomidine infusion for PSH management.

Our results present a nuanced picture of the comparative efficacy of these two treatment approaches. While the dexmedetomidine combination appeared to exert more pronounced effects on cardiovascular and respiratory parameters, the similarity in other clinical outcomes suggests that both treatments may offer viable options for PSH management, albeit with different physiological impacts.

Our selection of a subanesthetic KI as an adjuvant to the SGB warrants precise neurobiological contextualization, as ketamine is not traditionally recognized as a first‐line PSH agent in clinical guidelines. The pathophysiology of post‐TBI neurostorming is driven not only by peripheral sympathetic hyper‐reactivity but also by a profound loss of descending cortical inhibitory pathways, leading to a state of spinal and supraspinal NMDA‐mediated “wind‐up” and hyperexcitability. While an isolated cervical sympathetic trunk block interrupts peripheral sympathetic outflow to the cranial and thoracic vasculatures, the addition of low‐dose, subanesthetic ketamine serves as a central NMDA receptor antagonist. At these subanesthetic concentrations, ketamine suppresses central hyper‐responsiveness and enhances neural plasticity without triggering hyperadrenergic states or severe respiratory depression.

This unique mechanistic synergy directly expands upon the findings of Wang et al., who highlighted the utility of isolated right‐sided SGBs for acute PSH termination [11]. While Wang et al. established that blocking the sympathetic chain can significantly blunt plasma catecholamine surges, our data pushes this paradigm further by demonstrating that a dual‐targeted approach peripheral sympathetic disruption via local anesthetic block combined with central NMDA modulation via ketamine can manage systemic PSH parameters effectively. This creates a balanced autonomic baseline that matches the therapeutic recovery rates of continuous dexmedetomidine infusions while demonstrating distinct differences in hemodynamic safety profiles.

Our investigation yielded several key findings, patients receiving dexmedetomidine (D group) exhibited significantly lower MABP and HR compared to those receiving SGB with subanesthetic dose ketamine (SK group) throughout the study period, suggesting a sustained effect of the treatment.

Several factors may explain the lower MABP and HR in the D group in this study: Dexmedetomidine is a highly selective *α*2‐adrenergic agonist known for its sympatholytic effects. It reduces central sympathetic outflow, leading to decreased catecholamine release. In the context of managing neurostorms, our results regarding the cardiovascular effects of dexmedetomidine are consistent with and build upon other studies [16–18] conducted in related clinical settings. These studies showed that, in comparison to conventional sedation regimens, dexmedetomidine may be advantageous in lowering secondary brain injury, preserving hemodynamic stability, and minimizing symptoms of sympathetic hyperactivity in patients with moderate‐to‐severe TBI.

In contrast, ketamine, even at subanesthetic doses, is known to have sympathomimetic effects. It can provide analgesia and sedation, but typically does not lower MAP or HR to the same extent as dexmedetomidine. It can increase catecholamine levels, potentially counteracting some of the sympatholytic effects of the SGB. The SGB interrupts sympathetic innervation to the head, neck, and upper extremities [19].

Dexmedetomidine was linked to a higher incidence of bradycardia and hypotension than other sedatives, including ketamine, according to a meta‐analysis conducted by Shehabi et al. [20] examining different sedatives in critically sick patients. This corroborates our finding that the D group had lower HR and MABP.

The decreased respiratory rates may enable lower ventilator rates and better ventilation–perfusion matching for individuals on mechanical ventilation, hence lowering the risk of lung damage from ventilators. Lower RR may make it possible to wean patients off of mechanical breathing earlier [21]. This study showed that both groups were comparable in lowering RR at 30 min, 1, 2, 8, 16, and 48 h after initiating treatment.

Liu et al. [22] reported that dexmedetomidine’s profile is generally acceptable, with fewer respiratory depressions compared to traditional sedatives.

The duration of resolution of paroxysmal hypermyotonia and ICU stay were comparable between two groups with only four patients in D group compared to three patients in SK group who were not show significant improvement and both groups were comparable. Moreover, patients showed statistically and clinically significant comparable improvement of GCS grading in the same group and in both groups.

Soltani et al. [23] suggested that early dexmedetomidine utilization may lead to improved clinical outcomes, including reduced mortality and shorter hospital stays, for patients with moderate–severe TBI requiring mechanical ventilation.

Previous studies [24, 25] reported that the dexmedetomidine ability to reduce delirium and agitation positions it as a preferred option in ICU settings, potentially decreasing hospital stays and associated mortality.

Ketamine primarily functions as a noncompetitive antagonist of NMDA receptors, leading to increased glutamate release and enhanced neuroplasticity. This process involves various pathways, including signaling through brain‐derived neurotrophic factor (BDNF) [26].

### 4.1. Limitation

This study has several limitations, including a short 48‐h follow‐up and evaluating the interventions only after a neurostorm had occurred. Future research should investigate their potential prophylactic effects in high‐risk patients. Crucially, the lack of intracranial pressure (ICP) and cerebral perfusion pressure (CPP) monitoring limits the ability to confirm if dexmedetomidine‐induced MAP reductions compromised cerebral perfusion.

### 4.2. Conclusions

A unilateral SGB combined with subanesthetic ketamine improves overall neurological recovery and ICU length of stay in post‐TBI PSH compared to continuous dexmedetomidine. However, dexmedetomidine provides a faster reduction in MABP and offers high practical value due to its noninvasive, intravenous delivery.

**Nomenclature**
PSH: Paroxysmal sympathetic hyperactivity
GCS: Glasgow Coma Scale
ICU: Intensive care unit
MABP: Mean arterial blood pressure
TBI: Traumatic brain injury
SGB: Stellate ganglion block
KI: Ketamine infusion
ASA: American Society of Anesthesiologists
BMI: Body mass index
IV: Intravenous

## Author Contributions

Eslam Sobhy Al Maghawry Mohamed registration and conception of the study. Alshaimaa Abdel Fattah Kamel designed the study. Abdalla Mohamed Goda Mohamed collected data. Abd El‐Rahman Wael Abd El‐Rahman Ali Elmesallamy reviewed the final manuscript. Mohamed Gaber Mohamed and Marwa Mohamed Medhat analyzed the data. Michael Adel Shaker prepared the tables and figures. Omar A. Khashaba perform ultrasound image.

## Funding

There is no financial support or funding of the present study.

## Disclosure

All authors reviewed the final manuscript and approved it.

## Ethics Statement

This prospective, randomized clinical trial approved by Zagazig University’s Institutional Review Board (ZU‐IRB# 278‐1‐4‐2024).

## Consent

Informed written consent was obtained from the first‐degree relative or legal guardian before enrollment in the study. The study was performed in accordance with the regulations and guidelines of Helsinki Declarations.

## Conflicts of Interest

The authors have no conflicts of interest.

## Supporting Information

Additional supporting information can be found online in the Supporting Information section.

## Supporting information


**Supporting Information** Supporting Table 1: Paroxysmal sympathetic hyperactivity were diagnosed on the basis of diagnostic criteria proposed by Baguley et al. [12]. Supporting Table 2: Glasgow Coma Scale [13].

## Data Availability

The datasets during this study are available from the corresponding author upon reasonable request.
