# Supplementary material for: Stellate Ganglion Block Combined With Subanesthetic Ketamine Versus Dexmedetomidine for the Management of Paroxysmal Sympathetic Hyperactivity (PSH) After Traumatic Brain Injury: A Randomized Trial
Source: Crit Care Res Pract. 2026 Nov 4;2026:2779975. doi: 10.1155/ccrp/2779975 (PMC13732649; doi:10.1155/ccrp/2779975)
Supplement: Supplementary file 1 [file CCRP-2026-2779975-s001.docx]

| **Suppl. Table 1: Paroxysmal sympathetic hyperactivity were diagnosed on the basis of diagnostic criteria proposed by Louraoui et al*.*****[12]** | | | | | |
| --- | --- | --- | --- | --- | --- |
| Clinical feature scale (CSF) | | | | | |
|  | 0 | 1 | 2 | 3 | Score |
| Heart rate (beats per minute) | <100 | 100–119 | 120–139 | >140 |  |
| Respiratory rate (breaths per minute) | <18 | 18–23 | 24–29 | >30 |  |
| Systolic blood pressure (mmHg) | <140 | 140–159 | 160–179 | >180 |  |
| Temperature (°C) | <37 | 37–37.9 | 38–38.9 | >39 |  |
| Sweating | Nil | Mild | Moderate | Severe |  |
|  |  |  |  |  |  |
| Posturing during episode | Nil | Mild | Moderate | Severe |  |
|  | | | CSF subtotal | |  |
| Diagnosis likelihood tool (DLT) – scored as 1 if present | | | | | |
| Clinical features occur simultaneously | | | | |  |
| Episodes are paroxysmal in nature | | | | |  |
| Sympathetic over-reactivity to normally non-painful stimuli | | | | |  |
| Features persist for >3 consecutive days | | | | |  |
| Features persist for >2 weeks post-brain injury | | | | |  |
| Features persist despite treatment of alternative diagnosis | | | | |  |
| Medication administered to decrease sympathetic features | | | | |  |
| >2 episodes | | | | |  |
| Absence of parasympathetic features during episodes | | | | |  |
| Absence of other presumed cause of features | | | | |  |
| Antecedent acquired brain injury | | | | |  |
|  | | | DLT subtotal | |  |
| Combined total (CSF + DLT) | | | | |  |
|  | | Unlikely | | | <8 |
| PSH diagnostic likelihood | | Possible | | | 8–16 |
|  | | Probable | | | >17 |

**Suppl. Table 2: Glasgow Coma Scale [13].**

| Category | Patient Response / Criterion | Score |
| --- | --- | --- |
| Eye Opening (E) | Spontaneous | **4** |
|  | To spoken word / sound | **3** |
|  | To pressure / painful stimulus | **2** |
|  | None | **1** |
| Verbal Response (V) | Oriented | **5** |
|  | Confused conversation | **4** |
|  | Words (inappropriate) | **3** |
|  | Sounds (incomprehensible) | **2** |
|  | None | **1** |
| Motor Response (M) | Obeys commands | **6** |
|  | Localizes to pain | **5** |
|  | Normal flexion (withdrawal) | **4** |
|  | Abnormal flexion (decorticate posturing) | **3** |
|  | Extension (decerebrate posturing) | **2** |
|  | None | **1** |
